# Editorial Notes and Miscellany

**Published:** 1912-04

**Authors:** 


### Editorial Notes and Miscellany.

DOCTOR WANTED for country practice; fine location; Williamson county, Texas. Inquire of Dr. J. H. Petty, Taylor, Texas.

---

A ROUSING CONFERENCE ON TUBERCULOSIS will be held at Waco, Texas, April 16 and 17, inst., by the Southwestern Anti-Tuberculosis League. The Texas representative, R. J. Newton, has appointed over one hundred delegates from this State.

---

BATTLE & CO. have just issued No. 18 of the Dislocation charts, which completes the set. They will be sent free to physicians on request. Also Fracture and Tumor charts if desired.

---

ONE OF THE MOST dangerous menaces to health is the unsanitary outhouse commonly found on the grounds of county and village schools. A practical way to abate this nuisance may be had by sending to the United States Department of Agriculture for Bulletin No. 463.

---

THE BACTERIAL THERAPIST for April is full of good reading. A copy will be sent free on request by the publisher, Dr. G. H. Sherman, 419 St. Aubin Avenue, Detroit, mentioning this Journal. Dr. Sherman has produced a meningococcus serum which he sells at 25 cents per dose. See advertisement, front form, opposite first reading.

---

AS A RESULT of the work of the Rockefeller Sanitary Commission, the people are being aroused to the need of sanitary inspection of rural homes and schools. Public sentiment is now forming for an efficient county superintendent of health to devote his whole time to the medical examination of rural school children and the conservation of the life and the health of the people.

---

MENINGITIS.—Information on the subject of this little understood and therefore dreadful disease will, I know, be thankfully received by my readers. I reproduce elsewhere in this issue the "Technique of Administering the Serum," from the United States Public Health Reports, January 26, 1912, Vol. XXVII, No. 4. The entire pamphlet (by Dr. W. H. Frost, Passed Assistant Surgeon, P. H. and M. H. S.) can be obtained free on request by application to the department, mentioning this Journal.

---

THE OLD "AMERICAN PRACTITIONER AND NEWS," so long established and edited by David W. Yandell, the giant of Louisville, Ky., has changed hands and gone to New York. Professor John W. Wainwright, M. D., has purchased it; also the "New England

Medical Monthly," of Boston. He combines the two into one, giving it the title, "The American Practitioner, Incorporating the N. E. Medical Monthly," etc. The first issue appeared in March (ult.). Exchanges are requested to mail to 80 Washington Square, east, New York City.

---

Dr. A. S. Garrett's Candidacy for Congressman-at-Large.—It is gratifying to learn from a close friend of Dr. Garrett, who travels extensively, that the prospects for his election are good. The physicians throughout the State are solid for him. We want a physician—one like Dr. Garrett—in Congress. He can successfully refute the many misrepresentations and other malicious machinations that are constantly urged upon Congress in regard, for instance, to the objects of the Owen bill and the pure food laws. Let every reader of this do all in his power to land the accomplished scholar and gentleman in the House of Congress.

---

Remember the Date. The Texas State Medical Association will meet at Waco, May 7, 8 and 9. The usual reduced rates on railroads and at hotels have been secured, and a large attendance is expected. Details will be given in the association's journal for April. We learn that active preparations are being made by the several committees at Waco to give the association a rousing reception, and we may expect to have a royal good time. By the bye, I rise to remark that it will be the very best time in the world for ye subscribers to the "Red Back" to take along an extra greenback bill or some coin of the realm and pay up arrears and renew.

---

"Spiritualism," as defined by Texas Statutes, interpreted by Assistant Attorney General Funderburk and quoted in the case of Kiddleditch vs. Williams, is "a belief that the spirits of the dead can communicate with the living thro' the agency of persons called 'mediums.'" The practice of this vagary by so-called mediums is prohibited by law, and they are regarded as vagrants and are subject to treatment as such. The Assistant Attorney General gives the following opinion in response to a request of the Governor for a definition of the status of advertising "Spiritualists": "Any person advertising and maintaining himself in whole or in part as a Spiritualist, having such occupation prohibited by law, is not, in my opinion, subject to an occupation tax, it not being the policy of the law to license a prohibited act."

CURE FOR GOUT.—"A glass of brandy here," said Mr. Pickwick. The brandy was brought, and Mr. Weller, after pulling his hair to Mr. Pickwick, and nodding to Sam, jerked it down his capacious throat as if it had been a thimbleful.

"Well done, father," said Sam. "Take care, old fellow, or you'll have a touch of your old complaint, the gout."

"I've found a sov'rin' cure for that, Sammy," said Mr. Weller, setting down his glass.

"A sovereign cure for the gout?" said Mr. Pickwick, hastily producing his note-book. "What is it?"

"The gout, sir," replied Mr. Weller, "the gout is a complaint as arises from too much ease and comfort. If ever you're attacked with the gout, sir, jist you marry a widder as has got a good loud woice, with a decent notion of usin' it, and you'll never have the gout agin. It's a capital prescription, sir. I takes it reg'lar, and I'll warrant it to drive away any illness as is caused by too much jollity."

Having imparted this valuable secret, Mr. Weller drained his glass once more, produced a labored wink, sighed deeply and slowly retired.—*Pickwick Papers.*

---

DEATH OF DR. AMOS GRAVES, SR., OF SAN ANTONIO.—Dr. Graves was one of the most widely known physicians of Texas. He died at his home, after a brief illness, March 10, ult., aged 70 years. He is survived by his wife, son, Dr. Amos Graves, Jr., two daughters, Mrs. Smithie Graves, of San Antonio, Mrs. George W. Martin, of Fort McPherson, Ga., a brother, Judge Alexander Graves, of Missouri, and two sisters, Mrs. Foxworth, of Mississippi, and Mrs. Beaumont, of Vicksburg. Dr. Graves was born at Mt. Carmel, Miss., January 26, 1842; was educated at Danville, Ky., and at an early age joined the Fourth Mississippi Confederate Calvary, under Forrest; was badly wounded through the lungs in a charge, July 14, 1864, which caused him to retire from the army. At the close of the war he entered the University of Virginia and graduated in medicine in the famous medical department of that institution. He later took his degree at Tulane University. Dr. Graves practiced in Missouri from 1869 to 1874, and removed to San Antonio, where he resided and practiced medicine till the day of his death—thirty-six years. He was regarded as one of the most eminent surgeons in the Southwest, filling the position of chief surgeon and medical director of the Atlantic system of the Southern Pacific for sixteen years, and for twenty years chief surgeon of the San Antonio & Aransas Pass Railway. He was a man of affairs and wealthy.

---

### EVIDENTLY.

BY TEX O'REILLY.

You have heard of Edgar's "Raven,"
How that croaking, joking sable,
Found a roost o'er Edgar's chamber door;
And the poet at the table
Joshed this bird of plumage sable,
While the raven quoted quaintly,
"Nevermore!"

He descants, with awesome feeling,
How that shadow came a stealing,
Uncouthly silhouetted on the floor;
But, if the lamp was on the table,
How the deuce would it be able
To cast a shadow backward from the door?
"Nevermore!"

Of late I have been thinking
That perhaps old Ed. was drinking,
When he piped the grewsome shadow on the floor,
For a sober, decent raven,
Perched on such a lofty haven,
Would never drop his shadow from the door.
Nay, Ed., methinks 'twas bug juice—
Nothing more.

—*Presidio County Light.*

EDITORIAL COMMENT.—I file a brief for Edgar. "I deny the allegation and defy the alligator." I deny that the lamp was on the table, and challenge Tex to prove it. It is a pure assumption, unwarranted by the facts. Edgar said:

"His eyes had all the seeming of a demon's that is dreaming,
And the lamp light o'er him gleaming cast his shadow on the floor."

Observe: Ed. said: "The lamp light 'o'er him gleaming.'" If the lamp had been on a table, he would have said the lamp light o'er him stealing cast his shadow on the *ceiling*. Hence we are warranted in inferring that the lamp was swung from overhead, somewhere, and that Tex himself has been taking bug juice. Reversed and remanded.

